# Supplementary material for: Behaviour, Furnishing and Vertical Space Use of Captive Callimico (Callimico goeldii): Implications for Welfare
Source: Animals (Basel). 2023 Jun 29;13(13):2147. doi: 10.3390/ani13132147 (PMC10339928; doi:10.3390/ani13132147)
Supplement: Supplementary file 1 [file animals-13-02147-s001.zip › animals-2445456-supplementary.pdf]

Ethogram

Allo- specific interaction- interactions with another individual

Allo-grooming- Manual parting and picking through the pelage of another animal; often involves hairs of groomed animal being pulled between groomer's teeth as well as licking of hairs and skin. A bout ends if an animal moves >0.5m away from the other and grooming is stopped for 60 seconds

Clinging- arms held around and object, individual is stationary

Con-specific interaction- interaction with another individual of the same species

Feeding- consumption of food

Forage- searching for food

Grooming -Same as Allogroom, except individual grooms self. A bout ends if the animal stops grooming for 60 seconds

Locomotion -Climbing, running, or jumping along trees or walls of enclosure

Lying- Animal lays with knees and elbows fully bent, torso parallel with substrate if hands and feet are on the same substrate, ventrum may or may not be in contact with substrate, head upright; tail may be resting behind animal or pulled between legs with animal's body resting on it

Scanning- distinct sweeping movement of the head where gaze is apparent

Other- Any behaviour not included in ethogram

Out of sight- individual cannot be located

Information recorded at interval scan per individual

Vertical Zone -	1 2 3 4 (1 being highest 4 the ground) *	
Behaviour	Taken from ethogram	
Substrate/Furnishing	Trunk	upright natural wood structure-normally but not always a living tree
	Branch small <5cm<br>medium 5-10cm<br>large 10cm-20cm<br>x-large 20cm +	natural wood sizes are for guidance only. Natural variation means size will be recorded at scan
	Rope/vine	
	Wire Mesh	
	Platform/shelf small	<50cm wide includes nestbox *roof*
	Platform/shelf large	>50 cm
	Nestbox	
	Bark	
	Soil	
	Grass	
	Basket	
	Log	can be an upright stump or horizontal trunk
	Bamboo	
	Vegetation	dense area of growth which may be at any zone but is a network of leaves or fine growth that obscures or partially obscures an individual
	Structure	Elements of the enclosure which may include but is not limited to metal support poles, or other, man made supports and walls which are not otherwise included above
	Other	
Orientation	Horizontal<br>Vertical<br>Angle	Orientation is at the point of contact
Visitors	0<br>1-4<br>5-10<br>11+	
Proximity	less than 1m con specific<br>less than 1m allospecific<br>less than 1m visitor	

* additional zones can be added to allow recording of specific areas depending on collection configuration
